# Nineteenth Annual Report of the Managers of the New York State Lunatic Asylum

**Published:** 1862-05

**Authors:** 


Nineteenth Annual Report of the Managers of the New York State Lunatic Asylum. Transmitted to the Legislature, Feb. 5th, 1862. New York.

This is a neatly printed pamphlet of 39 pages, chiefly occupied with a statistical report of the Superintendent and Physician, Dr. John P. Gray. There is also a brief report from the Board of Managers and the Treasurer, with an appendix, containing the laws regulating the admission of patients, and the general management of the Institution.

